# Effect of catheter ablation combined with left appendage occlusion for non-valvular atrial fibrillation: a meta-analysis

**DOI:** 10.1186/s13019-022-01885-9

**Published:** 2022-05-31

**Authors:** Jun Qu, Zhen Wang, Shuhao Wang

**Affiliations:** 1 Department of Internal Medicine-Cardiovascular, YanTai YuHuangDing Hospital, Yantai, Shandong China; 2 Department of Internal Medicine-Cardiovascular, YanTai YuHuangDing Hospital, Yantai, Shandong China; 3 Department of Internal Medicine-Cardiovascular, LiaoCheng People’s Hospital, No. 67, Dongchang West Road, Liaocheng, 252000 Shandong China

**Keywords:** Ablation, Left appendage occlusion, Atrial fibrillation, Meta-analysis

## Abstract

**Objective:**

To estimate the effect of catheter ablation combined with left appendage occlusion in the treatment of non-valvular atrial fibrillation (NVAF) by a method of meta-analysis.

**Methods:**

Pubmed, Embase, and Cochrane Library were searched for the studies about catheter ablation combined with left appendage occlusion in treating NVAF. The data analysis was performed using R 4.0.5 software. The retrieval time was from inception to May 12, 2021.

**Results:**

A total of 18 published studies were identified in the meta-analysis, including 1385 participants. During the perioperative period of catheter ablation combined with left appendage occlusion in treating NVAF, the pooled incidences of pericardial effusion, major or minor bleeding events, and residual flow documented were 0.5%(95%CI 0.0002–0.0099), 1.42%(95% CI 0.00–0.04), 7.24%(95% CI 0.0447–0.0975), respectively. During the follow-up, the incidences of all-cause mortality, embolism events, and bleeding events were 0.32%(95%CI 0.0000–0.0071), 1.29%(95%CI 0.0037–0.0222), 2.07%(95% CI 0.0075–0.0339), respectively. In the follow-up period of the transesophageal echocardiography, the most complication was residual flow event with an incident rate of 10.80%(95% CI 0.054–0.1620). The maximum occurrence probability of residual flow events was 17.92% (95% CI 0.1133–0.2452). Moreover, the incident rate of NVAF recurrence was 29.23% (95% CI 0.2222–0.3623).

**Conclusion:**

The meta-analysis suggests that the “one-stop” procedure is effective for those patients undergoing NVAF. However, Patients with more residual blood flow have a higher incidence of bleeding complications. More studies are warranted to verify the safety and efficacy of catheter ablation combined with left appendage occlusion in terms of the “one-stop” procedure.

## Introduction

Atrial fibrillation (AF) is the most common clinical arrhythmia, especially in patients with structural heart disease [1, 2]. The interventional treatments of nonvalvular atrial fibrillation (NVAF) have made significant progress. However, the long-term recurrence rate of catheter ablation is high, and the evidence to reduce the risk of embolism is insufficient [1, 2]. As a minimally invasive interventional treatment, left atrial appendage occlusion can replace oral anticoagulants to prevent embolism in patients with NVAF, reducing the risk of bleeding caused by anticoagulants [1].

One-stop treatment, namely catheter ablation and left atrial appendage occlusion, was completed in a single hospitalization to achieve the combined intervention of stroke prevention and symptom treatment. AF increases the risk of stroke five-fold, associated with about 15% of strokes [1]. Anticoagulants can reduce the risk of ischemic stroke in patients with AF, but there are limitations. Catheter ablation is an effective approach for restoring and maintaining sinus rhythm in patients with AF, with the evidence to reduce the potential risk of stroke insufficient [2]. Even if there is no recurrence of AF after ablation, most guidelines still recommend preventive thrombolysis therapy [3, 4]. Left atrial appendage occlusion can replace oral anticoagulants to prevent embolism, reducing the risk of bleeding and mortality [5, 6]. Therefore, in theory, the combination of catheter ablation with left atrial appendage occlusion for one-stop treatment of NVAF can both restore sinus rhythm, reducing the risk of stroke caused by anticoagulant medication.

A number of studies [7–10] have proved the feasibility of one-stop treatment, forming a combination sequence of two one-stop treatments: ablation followed by occlusion, and occlusion followed by ablation.

The safe endpoints of left atrial appendage occlusion mainly included severe hemorrhage, pericardial effusion, and device embolism, which might be the outcomes due to the prolonged post-operation period. At present, the effects of catheter ablation combined with left appendage occlusion in the treatment of NVAF are still controversial. Therefore, this meta-analysis aims to explore the effect of catheter ablation combined with left appendage occlusion in the treatment of NVAF, to provide evidence of evidence-based medicine for the clinical treatment of NVAF.

## Methods

The meta-analysis was conducted according to the Preferred Reporting Items for Systematic Reviews and Meta-Analyses (PRISMA) [11].

### Literature screening

The databases containing Pubmed, Embase, and Cochrane Library were retrieved for relevant studies up to May 14, 2021. The retrieval strategy was as follows: (“Catheter ablation” OR “ablation”) AND (“Left atrial appendage occlusion” OR “Left appendage occlusion”) AND (“Atrial fibrillation” OR “NVAF” OR “Non-valvular atrial fibrillation”). The language was restricted to English. The literature search was carried out independently by two researchers and finally cross-checked. If there were disputes, they were resolved through discussion.

### Eligibility of the included literature

#### Inclusion criteria

(1) The type of study was clinical trial research; (2) The study subjects were patients with NVAF; (3) The intervention measure was catheter ablation combined with left atrial appendage occlusion; (4) The reported embolic complications in the retrieved studies included systemic embolism or ischemic stroke.

#### Exclusion criteria

(1) Experimental animal research, review, letter, and conference abstract; (2) Inadequate information on outcome indicators; (3) Duplicate data; (4) Literature not published in English.

### Quality assessment

Two researchers independently performed the quality evaluation of eligible studies following the Methodologic Index for Non-randomized Studies (MINORS) scales [12]. Only the non-comparative studies with more than 8 points and comparative studies with more than 12 points were included in this meta-analysis.

### Data extraction

The data extraction was performed independently by two researchers, and the consensus was reached after discussion. The following data were retrieved: (1) basic information: First author, year of publication, country, age and gender of the participants, sample size, the follow-up, the transesophageal echocardiography (TEE) follow-up, CHADS2, CHA2DS2-VASc, and HAS-BLED scores. (2) Complications happening during the perioperative period and follow-up period: bleeding, residual flow, embolic events, all-cause mortality, or NVAF recurrence during follow-up. Situations included any embolism, device-related thrombosis, and systemic thrombosis were combined into one category as “embolism events”.

### Statistical analysis

The data process was conducted by R 4.0.5 software. To summarize the outcomes, the results were expressed as an incident rate (number of events to number of patients) and 95% confidence interval (CI). Major complications such as bleeding, residual flow, embolic events, all-cause mortality, and NVAF recurrence during follow-up were analyzed. Since none of the studies provided exact data or standard deviations for the mean rate of events for each sample, the pooled mean did not show the standard deviation. I^2^ statistics and Cochran *Q* were used for the heterogeneous tests. If there was heterogeneity (I^2^ > 50%, or *p* < 0.1), the random-effects model (REM) was used for overall estimation. Otherwise, the fixed-effects model (FEM) was selected. Publication bias was evaluated by Egger's test. *p* < 0.05 was considered statistically significant.

## Results

### Literature screening and basic characteristics of the included studies

**Fig. 1:**
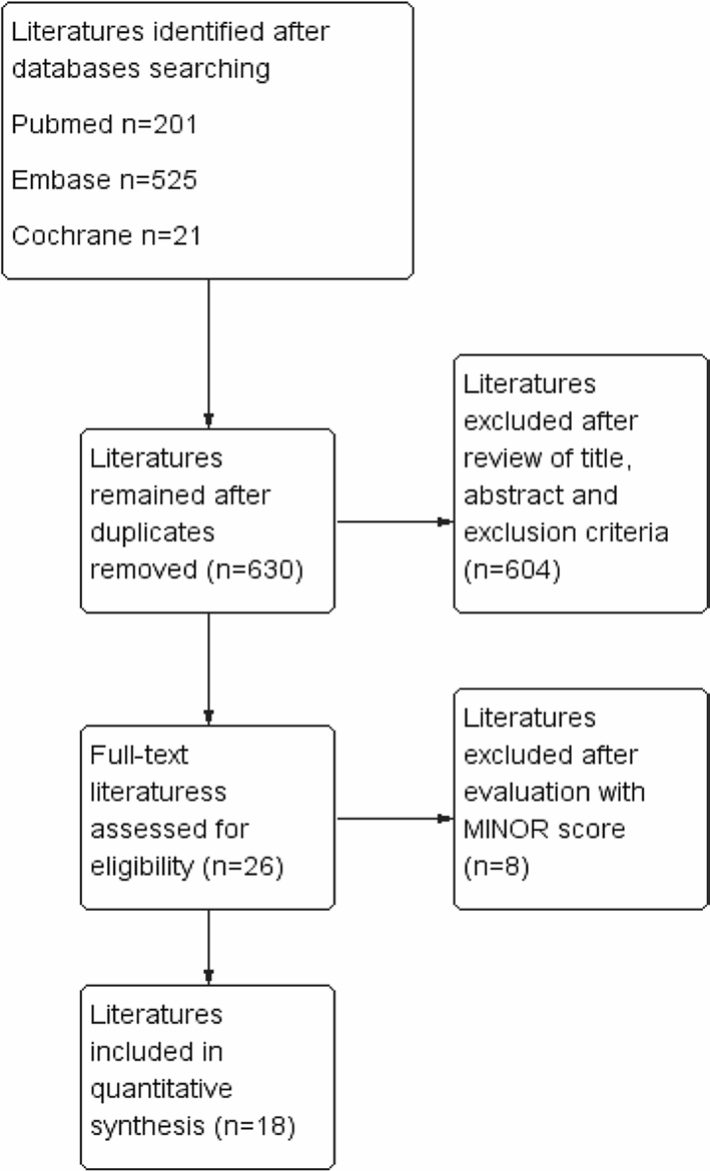
Flow chart of literature screening

After the first screening, a total of 747 articles were retrieved. After removing duplicated articles, 630 articles remained. Finally, after reading the full texts, a total of 18 studies were enrolled [7–10, 13–26], including 1385 participants. The flow chart of literature screening was shown in Fig. 1. The basic characteristics of the included literature were shown in Table 1. The follow-up was from 1 to 38 months, with an estimated average follow-up of 18.72 months. A total of 401 participants suffered from recurrent NVAF (Table 1). All the MINORS scores were greater than 12, indicating that all the studies included in this meta-analysis were of high quality.

**Table 1:** Basic characteristics of the included studies

Study/year	Sample size	Gender (male/female)	Age	Country	Follow-up (month)	TEE follow-up (month)	CHADS2	CHA2DS2-VASc	HAS-BLED	MINORS
Swaans 2012 [14]	30	21/9	62.8 ± 8.5	Netherlands	12	6	2.5	3	2	19
Walker 2012 [15]	26	20/6	63.0 ± 7.0	Australia	12	6	1.9	2.6	-	17
Swaans 2013 [16]	10	5/5	61.6 ± 9.6	Netherlands	6	1.5	3	3.5	1.5	13
Alipour 2015 [8]	62	40/22	64.0 ± 8.0	Netherlands	38	2	2.5	3.0	2	17
Calvo 2015 [7]	35	25/10	70.0 ± 7.0	Spain	13	3	2.01	3.1	3.1	16
Romanov 2015 [17]	45	28/17	60.0 ± 5.0	Russia	24	6	-	2.2	3.5	14
Phillips 2016 [9]	98	67/31	65.0 ± 7.0	Austrialia	26.73	12	1.5	2.6	1.9	15
Fassini 2016 [10]	35	28/35	72.0 ± 4.0	Italy	24	12	-	3	3	15
Panikker 2016 [18]	20	13/7	68.0 ± 7.0	United Kindom	12	9	-	3.1	2.5	16
Pelissero 2017 [19]	21	14/7	68.42 ± 10.61	Italy	14.93	14.93	-	2.8	3.2	15
Wintgens 2018 [13]	349	202/147	63.1 ± 8.2	Netherlands/Russia/Australia/Spain	34.5	3	2.0	3.0	3.0	15
Phillips 2018 [20]	139	76/139	64.1 ± 7.3	Australia/Russia/Netherlands/Italy/USA/Malaysia	1	1	2.2	3.4	1.5	17
Du 2018 [21]	82	48/34	66.2 ± 8.4	China	11.2	6	-	4.4	3.5	18
Li 2020 [22]	61	25/36	66.7 ± 9.2	China	24	12	-	4.2	3.4	19
Ren 2020 [23]	76	47/29	67.0 ± 7.5	China	24	12	-	3.4	2.3	16
Mo 2020 [24]	76	39/37	69.9 ± 7.9	China	24	6	-	3.6	3.3	18
Chen 2020 [25]	178	94/84	68.9 ± 8.1	China	12	3	-	3.3	1.6	17
Ren 2020 [26]	42	26/16	70.0 ± 7.6	China	20	12	-	3.8	3.7	17

TEE, transesophageal echocardiography. MINORS, methodological index for non-randomized studies

### Results of meta-analysis

#### Complications in the peri-operative period

**Fig. 2:**
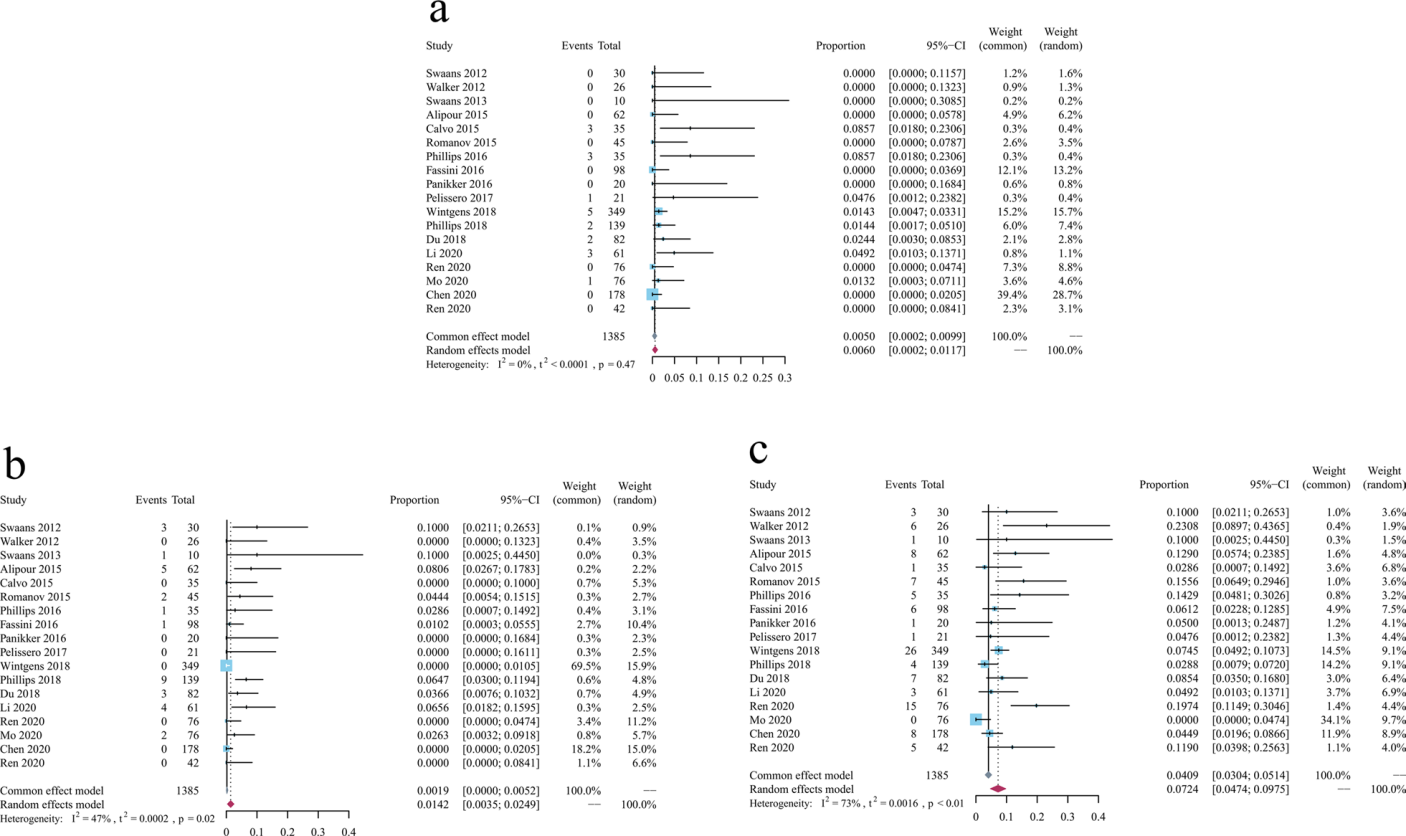
Forest plots of perioperative complications of NVAF treated by catheter ablation combined with left atrial appendage occlusion. **a** Pleural effusion; **b** Bleeding events; **c** Residual flow events. NVAF: nonvalvular atrial fibrillation

The results of the meta-analysis of catheter ablation combined with left atrial appendage occlusion in the treatment of NVAF showed that, during the peri-operative period, the pooled incidence of pericardial effusion (Fig. 2a) was 0.5% (95%CI 0.0002–0.0099), with FEM used (I^2^ = 0.0%, *p* = 0.47). The pooled incidence of major or minor bleeding events and residual flow documented (Fig. 2b) were 1.42% (95% CI 0.00–0.04), with REM selected (I^2^ = 47%, *p* = 0.02). Besides, the incidence of residual flow documented (Fig. 2c) was 7.24% (95% CI 0.0447–0.0975), with REM used (I^2^ = 73%, *p* < 0.01).

#### Complications in follow-up

**Fig. 3:**
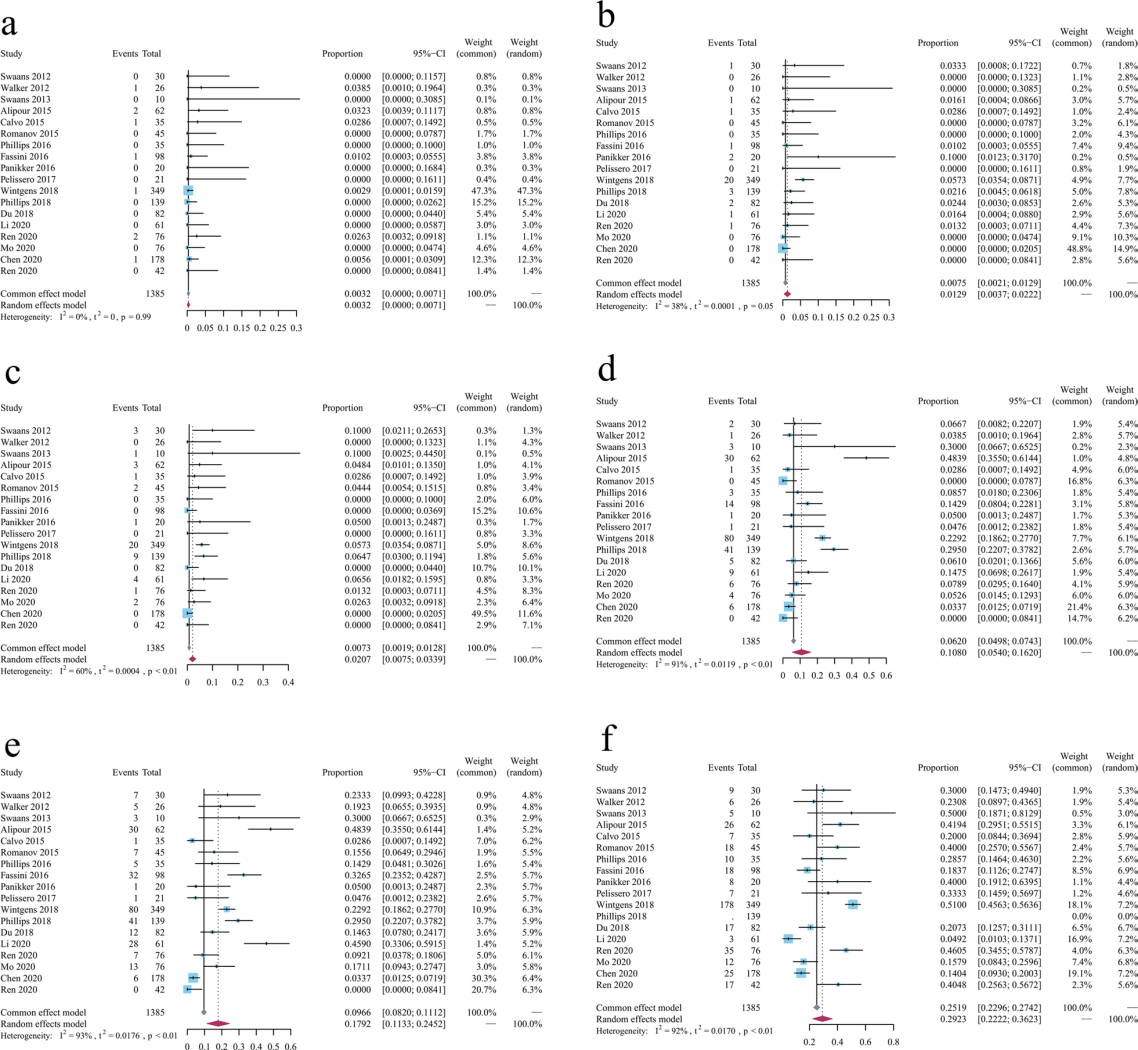
Forest plots of follow-up complications of NVAF treated by catheter ablation combined with left atrial appendage occlusion. **a** All-cause mortality; **b** Embolisms; **c** Bleeding events; **d** Residual flow events; **e** Maximum occurrence probability of residual flow events; **f** NVAF recurrence. NVAF: nonvalvular atrial fibrillation

The pooled incidence of all-cause mortality was 0.32% (95%CI 0.0000–0.0071), but no participants died due to pericardial effusion (FEM; I^2^ = 0.0%, *p* = 0.99) (Fig. 3a). The pooled incidence of embolism events (Fig. 3b) was 1.29% (95% CI 0.0037–0.0222) (I^2^ = 38%, *p* = 0.05; REM). In addition, the pooled incidence of bleeding events (Fig. 3c) was 2.07% (95% CI 0.0075–0.0339), with FEM adopted (I^2^ = 60%, *p* < 0.01).

In the follow-up period of the TEE, the pooled incidence of residual flow events (Fig. 3d) was 10.80% (95% CI 0.054–0.1620) (I^2^ = 91%, *p* < 0.01; REM). Maximum occurrence probability of residual flow events (Fig. 3e) was 17.92% (95% CI 0.1133–0.2452) (I^2^ = 93%, *p* < 0.01; REM). Moreover, the pooled AF recurrence incidence (Fig. 3f) was 29.23% (95% CI 0.20–0.38) (I^2^ = 92%, *p* < 0.01; REM).

### Publication bias detection

In the pooled analysis of pleural effusion, bleeding events, residual flow events, all-cause mortality, embolisms, follow-up bleeding events, follow-up residual flow events, maximum occurrence probability of residual flow events, and NVAF recurrence, the *p* values of the Egger’s test were 0.2248,0.0515,0.1840, 0.0549, 0.3866, 0.9449, 0.1673, 0.6950, 0.8280, respectively. This indicates that there is no significant publication bias in this meta-analysis.

## Discussion

Patients with NVAF have a significantly increased risk of stroke [27]. Left appendage occlusion became an alternative method to long-term anticoagulant therapy instead of warfarin or other anticoagulants. The efficacy of left appendage occlusion in preventing all-cause stroke was similar to that of warfarin [28].

In the "one-stop" treatment of NVAF, two steps of ablation with occlusion need to be completed at the same time. Therefore, it is inevitable to raise several questions. For instance, there are priority surgical issues of ablation and closure, as well as the possible debates about the better surgical procedure. A previous study reported the superiority of one-stop intervention [29]. At present, it is believed that for ablation followed by occlusion, the isolation of the vestibular lobe of the left pulmonary vein may ablate the ridge between the left superior pulmonary vein and the left atrial appendage. So, the edema and other effects generated in this position may affect the occlusion effect in the later stage of the occlusion device, leading to the occurrence of postoperative residual leakage. In this meta-analysis, we found residual flow events of 17.92% after the occlusion procedure, which was lower than the approximately 60% of persistent leak after left appendage occlusion. Besides, the extra risk for thrombosis could occur when the residual leaks were greater than 5 mm, then increasing the risk of stroke. Consequently, left appendage occlusion could be a potential risk for promoting stroke [30]. However, for the ablation after occluding, the existence of the occluder may bring inconvenience to the subsequent ablation of the crest, which may have a certain impact on the ablation effect.

Romanov et al. [17] enrolled 89 patients with parochial or persistent AF complicated with a high risk of thromboembolism and bleeding, and randomly assigned them to the ablation group and the Watchman + ablation group to evaluate whether the left atrial appendage occlusion affected the antiarrhythmic effect of NVAF ablation. Ninety-eight percent of the patients were implanted with an electrocardiograph to ensure continuous monitoring of heart rate and heart rhythm. The results showed that, although the load of NVAF increased significantly in the occlusion + ablation group during the 3-month blank period after surgery, the left atrial appendage occlusion did not affect the success rate of NVAF ablation compared with the ablation group alone at a follow-up of 24 months.

Singh et al. [31] evaluated the size and histological characteristics of the left atrial appendage in patients with AF ablation after pulmonary vein isolation. A total of 8 patients were included in this study and underwent contrast-enhanced MRI at 48 h and 3 months after ablation to compare the changes in these indicators. No significant changes in diameter, area, or histological characteristics of the left atrial appendage were observed preoperatively, 48 h postoperatively, or 3 months postoperatively in patients who underwent the first ablation of AF. The results of this study suggest that the ablation followed by occlusion may not affect the occlusion effect of the left atrial appendage, providing a theoretical basis for the ablation followed by occlusion.

A total of 82 patients with NVAF received a "one-stop" treatment of NVAF ablation combined with Watchman occlusion of the left atrial appendage. Fifty-two patients underwent occlusion followed by ablation, and the remaining 30 patients underwent radiofrequency followed by occlusion. The results showed that the two different sequences of surgical procedures were both safe and effective. However, the incidence of postoperative leakage around the occluder was lower in the occluding group followed by the ablation group during the follow-up. It suggested that the occluding procedure first might be better. The *p* values of the Egger’s test of all outcome indicators were greater than 0.05, which indicated that there was no significant publication bias in this study. However, these findings still need to be verified by further large-scale clinical studies.

In clinical practice, ablation can be performed with different energy, such as radiofrequency ablation, cryoablation, ultrasound ablation, laser ablation, etc. In addition to the "plug" type of Watchman, the left atrial appendage occluder also has the "cap" type such as ACP and Lambre. When these factors are taken into account and different permutations are generated, the choice of a "one-stop" treatment for NVAF becomes more complex. However, at the same time, these different surgical options also provide options for finding the best "one-step" procedure, which is also the main problem to solve in the future with the improvement of "one-stop" surgery for NVAF.

It is essential to note the "one-stop" management of indications for NVAF. The above study by Fauchier et al. [32] suggested that thromboembolism was not rare even if left atrial appendage occlusion was implemented in AF. In contrast, the low incidence of stroke after left atrial appendage occlusion in China suggests that most patients at low risk of stroke may be included in occlusion therapy. Therefore, the indications for "one-stop" treatment of NVAF should also be strictly grasped and implemented in patients with true indications of left atrial appendage occlusion to ensure that patients can truly benefit from it.

Therefore, the “one-stop” procedure is effective for those patients who underwent NVAF. However, NVAF Patients with higher residual blood flow have a higher incidence of bleeding complications. Certain studies compared the clinical outcomes between the two approaches, which showed decreases in postoperative complications. No antagonistic or potentially adverse interaction factors have been found in the reported studies of ablation and plugging. However, there are certain perioperative complications and adverse events reported in the literature. Therefore, it is reasonable to further investigate that the "one-stop" treatment of NVAF is safe and effective.

However, several limitations should be considered in this meta-analysis. First, some of the included studies had small participants unable to perform the analysis on large evidence bases. Second, the follow-up period varied differently to an obvious extent, which certainly caused the inaccuracy in the outcomes. Third, the majority of the included studies were performed without comparison, which was unable to compare different surgical approaches. Fourth, this meta-analysis only included articles published in English, excluding high-quality studies published in other languages, which might lead to a certain publication bias.

In conclusion, the "one-stop" treatment of NVAF ablation combined with left atrial appendage occlusion not only alleviates the symptoms of patients but also takes the prevention of stroke into account. It is an optimal possible interventional treatment reflecting the comprehensive management of NVAF under the current theoretical background and clinical practice conditions. Considering the limitations of this study, it is necessary to perform more studies focusing on the “one-stop” procedure to verify the safety and efficacy of catheter ablation combined with left appendage occlusion in treating NVAF in the future.

## Data Availability

The datasets used and/or analysed during the current study are available from the corresponding author on reasonable request.

## Abbreviations

AF: Atrial fibrillation
NVAF: Nonvalvular atrial fibrillation
VKAs: Vitamin K antagonists
PRISMA: Preferred Reporting Items for Systematic reviews and Meta-Analyses
MINORS: Methodologic index for nonrandomized studies
CI: Confidence intervals
TEE: Transesophageal echocardiography

## References

[1] Price MJ (2014). Left atrial appendage occlusion with the WATCHMAN™ for stroke prevention in atrial fibrillation. Rev Cardiovasc Med.

[2] Cappato R, Calkins H, Chen SA, Davies W, Iesaka Y, Kalman J (2010). Updated worldwide survey on the methods, efficacy, and safety of catheter ablation for human atrial fibrillation. Circ Arrhythm Electrophysiol.

[3] January CT, Wann LS, Alpert JS, Calkins H, Cigarroa JE, Cleveland JC (2014). 2014 AHA/ACC/HRS guideline for the management of patients with atrial fibrillation: a report of the American College of Cardiology/American Heart Association Task Force on Practice Guidelines and the Heart Rhythm Society. J Am Coll Cardiol.

[4] Kirchhof P, Benussi S, Kotecha D, Ahlsson A, Atar D, Casadei B (2017). 2016 ESC guidelines for the management of atrial fibrillation developed in collaboration with EACTS. Rev Esp Cardiol (Engl Ed).

[5] Holmes DR, Kar S, Price MJ, Whisenant B, Sievert H, Doshi SK (2014). Prospective randomized evaluation of the Watchman Left Atrial Appendage Closure device in patients with atrial fibrillation versus long-term warfarin therapy: the PREVAIL trial. J Am Coll Cardiol.

[6] Reddy VY, Doshi SK, Kar S, Gibson DN, Price MJ, Huber K (2017). 5-year outcomes after left atrial appendage closure: from the PREVAIL and PROTECT AF trials. J Am Coll Cardiol.

[7] Calvo N, Salterain N, Arguedas H, Macias A, Esteban A, García de Yébenes M (2015). Combined catheter ablation and left atrial appendage closure as a hybrid procedure for the treatment of atrial fibrillation. Europace.

[8] Alipour A, Swaans MJ, van Dijk VF, Balt JC, Post MC, Bosschaert MAR (2015). Ablation for atrial fibrillation combined with left atrial appendage closure. JACC Clin Electrophysiol.

[9] Phillips KP, Walker DT, Humphries JA (2016). Combined catheter ablation for atrial fibrillation and Watchman® left atrial appendage occlusion procedures: five-year experience. J Arrhythm.

[10] Fassini G, Conti S, Moltrasio M, Maltagliati A, Tundo F, Riva S, Dello Russo A, Casella M, Majocchi B, Zucchetti M (2016). Concomitant cryoballoon ablation and percutaneous closure of left atrial appendage in patients with atrial fibrillation. Europace.

[11] Moher D, Liberati A, Tetzlaff J, Altman DG (2009). Preferred reporting items for systematic reviews and meta-analyses: the PRISMA statement. PLoS Med.

[12] Slim K, Nini E, Forestier D, Kwiatkowski F, Chipponi J (2003). Methodological index for non-randomized studies (MINORS): development and validation of a new instrument. ANZ J Surg.

[13] Wintgens L, Romanov A, Phillips K, Ballesteros G, Swaans M, Folkeringa R (2018). Combined atrial fibrillation ablation and left atrial appendage closure: long-term follow-up from a large multicentre registry. Europace.

[14] Swaans MJ, Post MC, Rensing BJ, Boersma LV (2012). Ablation for atrial fibrillation in combination with left atrial appendage closure: first results of a feasibility study. J Am Heart Assoc.

[15] Walker DT, Humphries JA, Phillips KP (2012). Combined catheter ablation for atrial fibrillation and Watchman(®) left atrial appendage occlusion procedures: a single centre experience. J Atr Fibrillation.

[16] Swaans MJ, Alipour A, Rensing BJ, Post MC, Boersma LV (2013). Catheter ablation in combination with left atrial appendage closure for atrial fibrillation. J Vis Exp.

[17] Romanov A, Pokushalov E, Artemenko S, Yakubov A, Stenin I, Kretov E (2015). Does left atrial appendage closure improve the success of pulmonary vein isolation? Results of a randomized clinical trial. J Interv Card Electrophysiol.

[18] Panikker S, Jarman JW, Virmani R, Kutys R, Haldar S, Lim E, et al. Left atrial appendage electrical isolation and concomitant device occlusion to treat persistent atrial fibrillation: a first-in-human safety, feasibility, and efficacy study. Circ Arrhythm Electrophysiol. 2016;9(7). 10.1161/CIRCEP.115.003710 27406602

[19] Pelissero E, Giuggia M, Todaro MC, Trapani G, Giordano B, Senatore G (2017). Combined left atrial appendage percutaneous closure and atrial fibrillation ablation: a single center experience. G Ital Cardiol (Rome).

[20] Phillips KP, Pokushalov E, Romanov A, Artemenko S, Folkeringa RJ, Szili-Torok T (2018). Combining Watchman left atrial appendage closure and catheter ablation for atrial fibrillation: multicentre registry results of feasibility and safety during implant and 30 days follow-up. Europace.

[21] Du X, Chu H, He B, Wang B, Liu J, Feng M (2018). Optimal combination strategy of left atrial appendage closure plus catheter ablation in a single procedure in patients with nonvalvular atrial fibrillation. J Cardiovasc Electrophysiol.

[22] Li XX, Tian Y, Shi L, Wang YJ, Zeng LJ, Huang LH (2020). One-stop hybrid procedure combining catheter ablation and left atrial appendage closure increases long-term risk for adverse events in patients with atrial fibrillation. PACE.

[23] Ren Z, Zhang J, Wang S, Jia P, Li X, Zhang J (2020). Two-year outcome from combining cryoballoon ablation and left atrial appendage closure: CLACBAC study. Front Cardiovasc Med.

[24] Mo BF, Sun J, Zhang PP, Li W, Chen M, Yuan JL (2020). Combined therapy of catheter ablation and left atrial appendage closure for patients with atrial fibrillation: a case-control study. J Interv Cardiol.

[25] Chen M, Wang ZQ, Wang QS, Sun J, Zhang PP, Feng XF (2020). One-stop strategy for treatment of atrial fibrillation: feasibility and safety of combining catheter ablation and left atrial appendage closure in a single procedure. Chin Med J (Engl).

[26] Ren Z, Zhang J, Zhu M, Zhao D, Li S, Yang H (2020). Cryoablation combined with left atrial appendage closure: a safe and effective procedure for paroxysmal atrial fibrillation patients. Cardiol Res Pract.

[27] Lin HJ, Wolf PA, Kelly-Hayes M, Beiser AS, Kase CS, Benjamin EJ (1996). Stroke severity in atrial fibrillation. Framingham Study Stroke.

[28] Holmes DR, Reddy VY, Turi ZG, Doshi SK, Sievert H, Buchbinder M (2009). Percutaneous closure of the left atrial appendage versus warfarin therapy for prevention of stroke in patients with atrial fibrillation: a randomised non-inferiority trial. Lancet.

[29] Jiang Y, Li F, Li D, Cheng Y, Jia Y, Fu H (2020). Efficacy and safety of catheter ablation combined with left atrial appendage occlusion for nonvalvular atrial fibrillation: a systematic review and meta-analysis. PACE.

[30] Garcia-Villarreal OA. Surgical closure of the left atrial appendage basal considerations before attempting with occluder devices. J Surg. 2017;3(2).

[31] Singh SM, Jimenez-Juan L, Danon A, Bastarrika G, Shmatukha AV, Wright GA (2015). Magnetic resonance imaging of the left atrial appendage post pulmonary vein isolation: Implications for percutaneous left atrial appendage occlusion. J Arrhythm.

[32] Fauchier L, Cinaud A, Brigadeau F, Lepillier A, Pierre B, Abbey S (2018). Device-related thrombosis after percutaneous left atrial appendage occlusion for atrial fibrillation. J Am Coll Cardiol.

